# Completeness and timeliness of records of SARS due to COVID-19 in the SIVEP-Gripe System, Brazil 2020-2023

**DOI:** 10.1590/1980-549720260034

**Published:** 2026-07-31

**Authors:** Taina Barbie do Espírito Santo, Alexandra Crispim Boing

**Affiliations:** I Universidade Federal de Santa Catarina, Post-Graduate Program in Collective Health - Florianópolis (SC), Brazil.

**Keywords:** Covid-19, Severe acute respiratory syndrome, Health information systems, Data accuracy

## Abstract

**Objective:**

To evaluate the completeness and timeliness of records of severe acute respiratory syndrome (SARS) due to COVID-19 in the SIVEP-Gripe system in Brazil, from 2020 to 2023.

**Methods:**

This was a descriptive study based on secondary, public, and anonymized data from SIVEP-Gripe, comprising 2,200,796 SARS due to Covid-19 records. Sixty-six sociodemographic, clinical, hospitalization, outcome, and treatment variables were analyzed. Completeness was classified according to Romero and Cunha (2006). Timeliness was assessed through the intervals between symptom onset and notification, notification and data entry, and notification and case closure, considering historical surveillance parameters (7, 30, and 60 days, respectively).

**Results:**

There was a predominance of variables classified as having very poor completeness throughout the analyzed period, despite a slight improvement in the good and excellent completeness classifications between 2020 and 2023. Regional heterogeneity was observed; Amapá, Mato Grosso do Sul, Santa Catarina, and Paraná achieved higher proportions of satisfactory completion, whereas Tocantins, Pernambuco, Mato Grosso, Rondônia, and Alagoas had the worst performance. Between 2020 and 2023, the average notification time decreased from 13 to 7 days. Data entry was performed on time in over 90% of records in most states, while case closure remained around 86%. Nevertheless, the 90% target for timely notification was not achieved.

**Conclusion:**

Despite improvements in the timeliness of records, completeness remained unsatisfactory and uneven across states. These findings highlight the need to strengthen data entry practices and invest in continuous training and systematic monitoring to ensure more consistent data for surveillance and public health planning.

## INTRODUCTION

The COVID-19 pandemic had global impacts on health and the economy^1^, highlighting the need for information systems capable of supporting timely decision-making. According to official data from the Ministry of Health, more than 39 million cases of the disease were recorded by December 2023. Of these, 2 million were classified as severe, resulting in 714,000 deaths^2^.

In Brazil, surveillance for severe acute respiratory syndrome (SARS), implemented in 2009 following the influenza pandemic, became strategic during the COVID-19 emergency, as it enables the monitoring of hospitalizations and deaths associated with SARS-CoV-2^3^.

However, ensuring effective and continuous case monitoring requires high-quality, reliable, and accurately populated health information systems (HIS). These systems must contain complete, up-to-date data recorded with precision and timeliness to provide adequate support for planning, decision-making, and the implementation of actions by managers^4^ ^,^ ^5^ ^,^ ^6^.

In this regard, completeness and timeliness are essential attributes of information systems, directly related to their performance^7^ ^,^ ^8^. Completeness refers to the proportion of records in a system that contain values other than “unknown” or “null”, quantifying the extent to which information system records are complete. Timeliness, in turn, indicates the speed at which information is made available for decision-making and is crucial for immediate actions, such as interrupting disease transmission chains^9^.

The importance of data completeness and timeliness becomes even more evident in public health emergency contexts, such as the COVID-19 pandemic, where information quality directly impacts decision-making. National and international studies have highlighted weaknesses in information systems during health emergencies, including low completeness and delays in data availability^10^. In Brazil, research conducted during the pandemic evaluated record quality at the state level or within single-year timeframes^3^ ^,^ ^11^ ^,^ ^12^; however, analyses covering longer periods and on a national scope remain scarce.

Given this gap, the present study aimed to analyze the completeness and timeliness of records of SARS due to COVID-19 in the Influenza Epidemiological Surveillance Information System (SIVEP-Gripe) in Brazil between 2020 and 2023.

## METHODS

This was a descriptive study on the quality of records regarding SARS due to COVID-19. In Brazil, SARS notification is mandatory and carried out via the SIVEP-Gripe information system, which stores clinical, epidemiological, and outcome data entered by the notifying hospital. Since 2020, the system has undergone updates to incorporate advancing knowledge about COVID-19 and to meet epidemiological surveillance needs^13^.

The study population consisted of SARS records with a final classification of COVID-19, notified in Brazil between March 1, 2020, and December 31, 2023, according to the epidemiological calendar. Case definition was based on the “Final case classification” field, including cases recorded under option 5, “SARS due to COVID-19”. Data were obtained from a public, anonymized database available on the website https://opendatasus.saude.gov.br/dataset/, updated on January 29, 2024.

Record quality was assessed by analyzing the completeness and timeliness of notification, data entry, and case closure for COVID-19-related SARS records in Brazil. Sixty-six variables from the SARS notification form were included; they were selected as representative of key items within each category, covering sociodemographic information (sex, age, pregnancy status, race/color, education, occupation), clinical characteristics and symptoms (nosocomial case, flu syndrome outbreak, fever, cough, sore throat, dyspnea, respiratory distress, oxygen saturation <95%, diarrhea, vomiting, abdominal pain, fatigue, loss of smell and taste, other symptoms), pre-existing conditions (risk factors, cardiovascular disease, hematological disease, Down syndrome, liver disease, asthma, diabetes, neurological disease, lung disease, immunodeficiency, kidney disease, obesity, body mass index [BMI] details), immunization and treatment (COVID-19 vaccine, date of 1st dose, date of 2nd dose, influenza vaccine, antiviral use, specific antiviral, other, treatment start date), and hospitalization and outcome (admission date, state/federative unit of admission, municipality of admission, intensive care unit (ICU) admission, ICU entry and exit dates, use of ventilatory support, X-ray, CT scan, sample collection and result, case closure criteria, case outcome, discharge or death date, closure date). In 2020, 63 variables were analyzed, as the three variables related to COVID-19 vaccination were only incorporated into the system in subsequent years.

To calculate the completeness of each variable, the numerator used was the number of COVID-19-related SARS records in which the field was properly filled out (i.e., not marked as “unknown” or “null”), while the denominator was the total number of COVID-19-related SARS records. The result was multiplied by 100 to obtain relative frequency. Completeness quality was classified according to adapted Romero and Cunha criteria^14^, which categorize completeness levels as excellent (>95%), good (91-95%), fair (71-90%), poor (50-70%), and very poor (<50%). Subsequently, the proportion of variables in each classification category was calculated.

Notification timeliness refers to the speed with which a case is reported to health authorities; it is assessed based on the time interval (in days) between the date of symptom onset and the date of notification. This is a crucial indicator of the surveillance system’s agility in enabling rapid and effective control measures^15^.

Timeliness of data entry refers to the speed of inputting data into the system, evaluated by the time interval between the notification date and the data entry date. Timeliness of case closure refers to the timeframe for completing the investigation and recording the final classification (confirmed/discarded) and outcome (recovery/death) of a notified case, measured by the difference between the notification date and the record closure date. The parameters adopted for timeliness were those historically used by epidemiological surveillance, in accordance with the Manual of Standards and Routines for the Notifiable Diseases Information System (Sinan). Accordingly, a notification was considered timely if made within seven days of symptom onset, data entry if performed within 30 days of notification, and case closure if completed within 60 days of the notification date^16^.

To calculate the percentage of timely notifications, data entries, and case closures, the number of records made within the timeframe defined as timely was used as the numerator, while the total number of COVID-19-related SARS records served as the denominator. The Ministry of Health establishes a benchmark requiring that more than 90% of records be notified and closed in a timely manner^17^.

Regarding the analysis of notification time, no specific exclusion of extreme or inconsistent values ​​was carried out; the data were analyzed exactly as provided in the database. It should be noted that, as the data were secondary, anonymized, and in the public domain, it was not possible to conduct an in-depth cleaning of the dataset, such as identifying duplicates or correcting potential individual inconsistencies.

Data extraction and descriptive statistical analysis were performed using R software, version 4.1.2. As the confidentiality and privacy of the information were ensured, there was no need to submit the study to a research ethics committee, in accordance with National Health Council Resolution No. 510/2016, which waives the requirement for ethical review for research using publicly accessible information.

## Data availability statement:

The data used in the study are derived from a public, anonymized database available on OpenDataSUS, as described in the Methods section (“The data were obtained from a public, anonymized database available on the OpenDataSUS website, updated on January 29, 2024”). Additionally, the entire dataset supporting the results of this study is available upon request to the corresponding author.

## RESULTS

Between 2020 and 2023, 2,200,796 records of SARS due to COVID-19 in Brazil were analyzed: 1,198,507 in 2020, 4,294,934 in 2021, 1,568,476 in 2022, and 161,459 in 2023. A predominance of variables classified as having “very poor” completeness was observed throughout the analyzed period, although there was a progressive reduction from 38.1% in 2020 to 30.3% in 2023. Conversely, variables classified as having a “good” level of completeness increased from 7.9 to 12.1% during the same period (Table 1). The highest volume of notifications occurred in 2021, followed by a sharp decline in subsequent years; however, high proportions of incomplete data persisted in the records.

**Table 1.** Percentage of variables for cases of COVID-19-related severe acute respiratory syndrome according to level of completeness and year of notification in Brazil, 2020-2023.

Level of completeness	2020		2021		2022		2023	
	%	n (63)	%	n (66)	%	n (66)	%	n (66)
Excellent	17.5	11	18.2	12	16.7	11	15.2	10
Good	7.9	5	3.0	2	9.1	6	12.1	8
Fair	25.4	16	19.7	13	19.7	13	18.2	12
Poor	11.1	7	22.7	15	22.7	15	24.2	16
Very poor	38.1	24	36.4	24	31.8	21	30.3	20

Source: SIVEP-Gripe, January 29, 2024.

**Figure 1.**
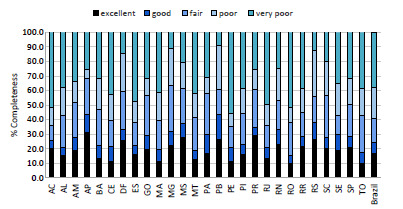
Percentage of variables of cases of COVID-19-related severe acute respiratory syndrome according to level of completeness and federative unit of residence, Brazil, 2020-2023.

Source: SIVEP-Gripe, January 29, 2024.

The states of Amapá, Mato Grosso do Sul, Santa Catarina, and Paraná showed the highest proportions of variables classified as “good” and “excellent”, collectively achieving a satisfactory completion rate of approximately 40 to 45%. In contrast, Tocantins, Pernambuco, Mato Grosso, Rondônia, and Alagoas exhibited the poorest performance, with a predominance of “poor” and “very poor” categories exceeding 50%. Nationally, it was observed that approximately 30% of the variables remained at unsatisfactory levels (“poor” and “very poor”) (Figure 1).

**Figure 2.**
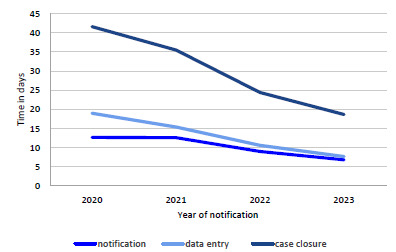
Average time interval (in days) between notification, data entry, and case closure for severe ccute respiratory syndrome due to COVID-19 in Brazil, 2020-2023.

Source: SIVEP-Gripe, January 29, 2024.

The analysis of timeliness (Figure 2) showed a significant reduction in the average processing intervals for records. The time between symptom onset and notification decreased from 13 to 7 days. Similarly, the average intervals for data entry and case closure were reduced by more than 50%, demonstrating greater efficiency in the record workflow and the finalization of case forms.

**Figure 3.**
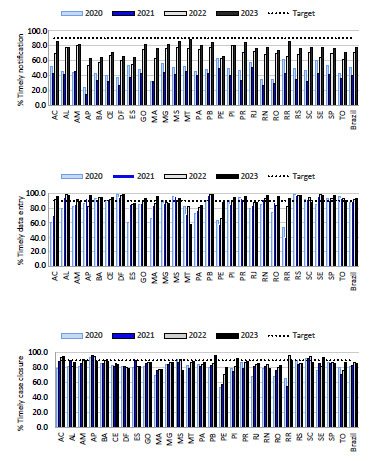
Percentage of timely notification, data entry, and case closure for severe acute respiratory syndrome due to COVID-19, by Brazilian federative unit, 2020-**2023.**

Source: SIVEP-Gripe, 29/01/2024.

Figure 3 presents the percentage of records notified, entered, and closed in a timely manner across Brazilian states. In 2020, only 50% of COVID-19-related SARS records in the country were notified within seven days of symptom onset. By 2023, this figure had risen to 76.9%. Mirroring the national trend, all states showed improvement in timely notification. Despite these advances, no state met the target of having 90% of cases notified within the recommended timeframe.

The assessment of data entry timeliness in Brazil revealed that, in 2020, over 87% of case forms were entered within 30 days of the notification date, a figure that rose to 93% in 2023. Most states increased their proportion of timely data entry over the years, meeting the 90% target set by the Ministry of Health. However, Espírito Santo, Mato Grosso, Pará, Pernambuco, and Rio de Janeiro were the states that failed to meet this benchmark (Figure 3).

Regarding timely closure within 60 days of the notification date, it was observed that during the analyzed period, approximately 86% of cases in Brazil were closed in a timely manner, with little variation across the years. The states that closed more than 90% of cases on time were Acre, Amapá, Mato Grosso do Sul, Paraíba, Piauí, Roraima, Santa Catarina, and Sergipe. Concerning timeliness targets, only the goal for timely data entry was met in 2022 and 2023, while the others remained below the established benchmarks (Figure 3).

## DISCUSSION

In this study, we analyzed the completeness and timeliness of COVID-19-related SARS records in the SIVEP-Gripe system. Completeness was found to be unsatisfactory, with 59% of variables classified as “poor” or “very poor”. Conversely, there was a significant reduction in the time taken for notification, data entry, and case closure, indicating greater speed in the system’s data input process. These findings corroborate previous studies^3^ ^,^ ^12^ that also identified low completeness in SARS records. Although the decrease in the number of records in 2022 and 2023 accelerated notification and closure processes, there was no proportional improvement in the quality of data entry, suggesting that operational speed was not matched by improvements in data consistency.

Heterogeneity was observed across federative units, with states such as Amapá, Mato Grosso do Sul, Santa Catarina, and Paraná showing better completeness levels, while others such as Tocantins, Pernambuco, and Rondônia showed unsatisfactory performance. This suggests structural inequalities in the organization of epidemiological surveillance. These differences may reflect variations in technological infrastructure, staff training, and operational recording workflows, including the use of paper forms followed by manual data entry. Previous studies also indicate that regional inequalities directly impact the quality of health information systems^7^. It should be noted that SIVEP-Gripe is a complex system involving multiple variables and operational instabilities; it underwent successive updates during the pandemic, which may have affected record quality. Furthermore, access to the system is not universal across health services, a factor that may influence the timeliness of notifications.

Low data completeness may be linked to operational and organizational factors, such as heavy workloads during critical pandemic periods, the prioritization of patient care over detailed data recording, and the perception of reporting as merely a bureaucratic task. Furthermore, a lack of understanding regarding the epidemiological importance of certain variables can contribute to incomplete form filling^15^. These factors are particularly significant given the substantial rise in notifications between 2020 and 2021, a period when the system faced immense demand.

Regarding timeliness, a significant reduction was observed in the average time between symptom onset and notification, dropping from 13 to 7 days between 2020 and 2023, alongside improvements in data entry and case closure intervals. These findings indicate that epidemiological surveillance services progressively adapted throughout the pandemic, achieving greater agility in information processing. However, despite this progress, the target of 90% timely notifications was not met in any federative unit, highlighting persistent limitations in the system’s capacity for timely response.

Similarly, while data entry rates exceeded 90% in most states in recent years, the rate of case closures remained around 86%, falling short of the recommended benchmark. This result suggests that more complex stages of the process, such as case investigation and finalization, continue to challenge surveillance services, even after the system became well-established during the pandemic.

At the onset of the health crisis, pandemic management required rapid responses and the adaptation of information systems for real-time data collection, analysis, and dissemination, elements essential for understanding the epidemiological landscape and making informed decisions. To achieve this, the data needed to be complete, reliable, and timely^18^ ^,^ ^19^.

During the early years of the pandemic, reporting delays may have been exacerbated by limitations within SIVEP-Gripe - which initially lacked specific fields for COVID-19 - as well as frequent system instability and a lack of interoperability with other systems, such as the Laboratory Environment Management System. Compounding this issue was the lack of systematic training for the professionals responsible for filling out the reporting forms^7^.

The observed poor data quality may be linked to periods of inaccessibility regarding SIVEP-Gripe data, often referred to as “data blackouts”. Notable instances include the suspension of the Coronavirus dashboard in June 2020 for political reasons, as well as a subsequent period of inaccessibility in December 2021 that lasted at least 30 days. Against this backdrop, the federal government proposed arbitrary changes to variable measurements and reporting standards that were inconsistent with international epidemiological guidelines^20^. Such events can compromise the generation of reliable indicators and the capacity for rapid response, while also hindering the identification of significant events and impeding the formulation of appropriate recommendations^21^.

This study enabled a comprehensive analysis of COVID-19 data quality in Brazil over four years of the pandemic, providing insights to improve record-keeping in the SIVEP-Gripe system. While the study yields important findings for surveillance and the assessment of health information quality, it has limitations: the analysis was restricted to confirmed cases rather than the full set of cases recorded in the system, and the dataset was not cleaned to remove duplicates or inconsistencies because of lack of access to individual-level data.

A key strength is the novel joint analysis of record completeness and timeliness, providing evidence to support proposals for improving data entry and system operations. A SIVEP-Gripe system that yields higher-quality, timely data contributes to effective territorial monitoring and more informed public health decision-making.

Data recording, processing, and analysis within HIS still face challenges. According to Melo and Soares^6^, difficulties associated with HIS include the lack of policies incentivizing accurate data entry, which hinders the generation of correct information and, consequently, the implementation of effective measures^22^.

The results indicate that, although processes have become faster, this has not been matched by consistent improvements in the quality of data entry. This underscores the need to implement periodic record assessment routines and strategies to enhance information quality within epidemiological surveillance services.

Despite the observed limitations, SIVEP-Gripe stands out as a fundamental system for epidemiological surveillance, particularly during emergencies. As one of the few online systems offering rapid data access, it enables timely analysis and supports decision-making. This feature distinguishes it from other systems, such as Sinan Net, which while equally relevant involves less agile workflows for information availability.

Furthermore, it is essential to establish continued training processes for the professionals involved in reporting to ensure the standardization and reliability of the information. Policies for ongoing training, periodic evaluations, active supervision, and systematic monitoring of data quality are crucial for reducing regional disparities and ensuring reliable data, which are indispensable for planning public health actions.
