# Supplementary material for: Completeness and timeliness of records of SARS due to COVID-19 in the SIVEP-Gripe System, Brazil 2020-2023
Source: Rev Bras Epidemiol. 2026 Jul 31;29:e260034. doi: 10.1590/1980-549720260034 (PMC13427215; doi:10.1590/1980-549720260034)
Supplement: Translation

# Translation: Completude e oportunidade dos registros de síndrome respiratória aguda grave por COVID-19 no Sistema de Informação da Vigilância Epidemiológica da Gripe, Brasil 2020-2023

**DOI:** 10.1590/1980-549720260034.2

**Authors:** Taina Barbie do Espírito Santo, Alexandra Crispim Boing

**Affiliations:** I Universidade Federal de Santa Catarina, Programa de Pós-Graduação em Saúde Coletiva - Florianópolis (SC), Brasil.

**Keywords:** COVID-19, Síndrome respiratória aguda grave, Sistemas de informação em saúde, Qualidade dos dados

## Abstract

**Objetivo:**

Analisar a completude e a oportunidade dos registros de Síndrome Respiratória Aguda Grave (SRAG) por COVID-19 no Sistema de Informação da Vigilância Epidemiológica da Gripe (SIVEP-Gripe), no Brasil, entre 2020 e 2023.

**Métodos:**

Estudo descritivo, baseado em dados secundários públicos e anonimizados do SIVEP-Gripe, referentes a 2.200.796 registros de SRAG por COVID-19. Foram analisadas 66 variáveis sociodemográficas, clínicas, de hospitalização, evolução e tratamento. A completude foi classificada segundo critérios de Romero e Cunha (2006). A oportunidade foi medida pelos intervalos entre início dos sintomas e notificação, notificação e digitação, e notificação e encerramento, considerando parâmetros históricos da vigilância (7, 30 e 60 dias, respectivamente).

**Resultados:**

Observou-se predominância de variáveis classificadas com completude muito ruim ao longo do período analisado, apesar de discreta melhora nas classificações boa e excelente entre 2020 e 2023. Houve heterogeneidade regional: Amapá, Mato Grosso do Sul, Santa Catarina e Paraná obtiveram maiores proporções de preenchimento satisfatório, enquanto Tocantins, Pernambuco, Mato Grosso, Rondônia e Alagoas tiveram pior desempenho. Entre 2020 e 2023, o tempo médio de notificação reduziu de 13 para 7 dias. A digitação superou 90% de registros oportunos na maioria dos estados, enquanto o encerramento permaneceu em torno de 86%. Ainda assim, a meta de 90% de notificações oportunas não foi alcançada.

**Conclusão:**

Apesar dos avanços na agilidade dos registros, a completude manteve-se insatisfatória e desigual entre estados. Esses achados evidenciam a necessidade de aprimorar o preenchimento das fichas e investir em capacitação e monitoramento sistemático, a fim de assegurar dados mais consistentes para a vigilância e o planejamento em saúde.

## INTRODUÇÃO

A pandemia da COVID-19 gerou impactos globais na saúde e na economia^1^, evidenciando a necessidade de sistemas de informação capazes de subsidiar decisões oportunas. Segundo os dados oficiais do Ministério da Saúde, até dezembro de 2023, foram contabilizados mais de 39 milhões de casos da doença. Entre esses casos, 2 milhões foram classificados como graves, resultando em 714 mil óbitos^2^.

No Brasil, a vigilância da Síndrome Respiratória Aguda Grave (SRAG), implantada em 2009 após a pandemia de influenza, tornou-se estratégica durante a emergência da COVID-19, pois permite monitorar hospitalizações e óbitos relacionados ao SARS-CoV-2^3^.

Entretanto, para assegurar um acompanhamento efetivo e contínuo dos casos, é fundamental dispor de Sistemas de Informação em Saúde (SIS) que sejam de qualidade, confiáveis e devidamente preenchidos. Esses sistemas devem conter dados completos, atualizados e registrados com precisão e oportunidade, de modo a oferecer suporte adequado ao planejamento, à tomada de decisões e à execução de ações por parte dos gestores^4^ ^,^ ^5^ ^,^ ^6^.

Nesse sentido, a completude e a oportunidade configuram atributos essenciais dos sistemas de informação, estando diretamente relacionadas ao seu desempenho^7^ ^,^ ^8^. A completude refere-se à proporção com que cada registro de um sistema apresenta valores distintos das categorias “ignorado” ou “nulo”, quantificando em que medida os registros de um sistema de informação estão completos. Já a oportunidade indica a velocidade com que as informações são disponibilizadas para a tomada de decisão e torna-se fundamental para ações imediatas, como a interrupção da cadeia de transmissão de doenças^9^.

A importância da completude e da oportunidade dos dados torna-se ainda mais evidente em contextos de emergência em saúde pública, como a pandemia de COVID-19, em que a qualidade da informação impacta diretamente a tomada de decisão. Estudos nacionais e internacionais têm apontado fragilidades nos sistemas de informação em contexto de emergências sanitárias, incluindo baixa completude e atrasos na disponibilização de dados^10^. No Brasil, pesquisas durante a pandemia avaliaram a qualidade dos registros em recortes estaduais ou limitados a um único ano^3^ ^,^ ^11^ ^,^ ^12^, mas ainda são escassas as análises que contemplam períodos mais longos e abrangência nacional.

Diante dessa lacuna, o presente estudo tem como objetivo analisar a completude e a oportunidade dos registros de SRAG por COVID-19 no Sistema de Informação da Vigilância Epidemiológica da Gripe (SIVEP-Gripe), no Brasil, entre 2020 e 2023.

## MÉTODOS

Trata-se de um estudo descritivo sobre a qualidade dos registros de SRAG por COVID-19. No Brasil, a notificação de SRAG é compulsória e realizada no sistema de informação SIVEP-Gripe, o qual armazena dados clínicos, epidemiológicos e de evolução, preenchidos pela instituição hospitalar notificadora. Desde 2020, o sistema passou por atualizações para incorporar o avanço do conhecimento sobre a COVID-19 e atender às demandas da vigilância epidemiológica^13^.

A população do estudo foi composta pelos registros de SRAG com classificação final COVID-19, notificados no Brasil entre 1º de março de 2020 e 31 de dezembro de 2023, segundo calendário epidemiológico. A definição dos casos considerou o campo “Classificação final do caso”, sendo incluídos aqueles registrados na opção 5, “SRAG por COVID-19”. Os dados foram obtidos de base pública, anonimizada, disponível no site https://opendatasus.saude.gov.br/dataset/, com atualização em 29 de janeiro de 2024.

A qualidade dos registros foi avaliada por meio da análise da completude e da oportunidade de notificação, digitação e encerramento dos registros de SRAG por COVID-19 no Brasil. Foram incluídas 66 variáveis da ficha de notificação de SRAG, selecionadas por representarem as principais de cada categoria, abrangendo informações sociodemográficas (sexo, idade, gestação, raça/cor, escolaridade, ocupação), características clínicas e sintomas (caso nosocomial, surto de Síndrome Gripal - SG, febre, tosse, dor de garganta, dispneia, desconforto respiratório, saturação abaixo de 95%, diarreia, vômito, dor abdominal, fadiga, perda de olfato e de paladar, outros sintomas), condições preexistentes (fatores de risco, doença cardiovascular, hematológica, Síndrome de Down, doença hepática, asma, diabetes, doença neurológica, pneumopatia, imunodeficiência, doença renal, obesidade, descrição do Índice de Massa Corporal - IMC), imunização e tratamento (vacina contra COVID-19, data da 1^a^ dose, data da 2^a^ dose, vacina da gripe, antiviral, qual antiviral, outro, início do tratamento), hospitalização e evolução (data da internação, Unidade Federativa - UF de internação, município de internação, internação em Unidade de Terapia Intensiva - UTI, data de entrada e saída da UTI, uso de suporte ventilatório, raio-X, tomografia, coleta e resultado, critério de encerramento, evolução do caso, data da alta ou óbito, data do encerramento). Em 2020, foram analisadas 63 variáveis, pois as 3 relacionadas à vacinação contra a COVID-19 foram incorporadas ao sistema apenas nos anos subsequentes.

Para o cálculo da completude de cada variável, considerou-se como numerador o número de registros de SRAG por COVID-19 em que o campo foi devidamente preenchido (diferente de “ignorado” ou “nulo”); e, como denominador, o total de registros de SRAG por COVID-19. O resultado foi multiplicado por cem para obtenção da frequência relativa. A qualidade da completude foi classificada segundo os critérios de Romero e Cunha adaptados^14^, os quais consideram a completude como percentual de preenchimento excelente (>95%), bom (91-95%), regular (71-90%), ruim (50-70%) e muito ruim (<50%). Em seguida, calculou-se a proporção de variáveis em cada categoria de classificação.

A oportunidade de notificação refere-se à rapidez com que um caso é notificado às autoridades de saúde, sendo avaliada pelo intervalo de tempo, em dias, entre a data de início dos sintomas e a data de notificação. É um indicador crucial da agilidade do sistema de vigilância para permitir ações de controle rápidas e eficazes^15^.

A oportunidade de digitação refere-se à agilidade na inserção dos dados no sistema, sendo avaliada pelo intervalo de tempo entre a data de notificação e a data de digitação. A oportunidade de encerramento refere-se ao tempo hábil para finalizar a investigação e registrar a classificação final (confirmação/descarte) e evolução (cura/óbito) de um caso notificado, sendo medida pela diferença entre a data da notificação e a data de encerramento dos registros.

Adotaram-se, como parâmetros de oportunidade, aqueles historicamente utilizados pela vigilância epidemiológica, conforme o Manual de Normas e Rotinas do Sistema de Informação de Agravos de Notificação (Sinan). Dessa forma, considerou-se oportuna a notificação realizada em até sete dias após o início dos sintomas, a digitação em até 30 dias após a notificação e o encerramento oportuno em até 60 dias a partir da data da notificação^16^.

Para o cálculo do percentual de notificações, digitações e encerramentos oportunos, considerou-se como numerador o número de registros realizados no prazo definido como oportuno, e como denominador, o total de registros de SRAG por COVID-19. O Ministério da Saúde estabelece como parâmetro que mais de 90% dos registros sejam notificados e encerrados oportunamente^17^.

No que se refere à análise do tempo de notificação, não foi realizada exclusão específica de valores extremos ou inconsistentes, sendo os dados analisados conforme disponibilizados na base. Ressalta-se que, por se tratar de dados secundários, anonimizados e de domínio público, não foi possível realizar uma limpeza mais aprofundada do banco, incluindo a identificação de duplicidades ou correção de possíveis inconsistências individuais.

A extração do banco de dados e a realização da estatística descritiva foi realizada no software R, versão 4.1.2. Por garantir o sigilo e a privacidade das informações, não houve necessidade de submissão do estudo ao Comitê de Ética em Pesquisa, em conformidade com a Resolução do Conselho Nacional de Saúde n^o^ 510/2016, que prevê a dispensa de apreciação ética para pesquisas que utilizam informações de acesso público.

## Declaração de disponibilidade de dados:

Os dados utilizados no estudo são provenientes de base pública, anonimizada, disponível no OpenDataSUS, conforme descrito na seção de Métodos (“Os dados foram obtidos de base pública, anonimizada, disponível no site OpenDataSUS, com atualização em 29 de janeiro de 2024”). Adicionalmente, todo o conjunto de dados que dá suporte aos resultados deste estudo está disponível mediante solicitação ao autor correspondente.

## RESULTADOS

No período de 2020 a 2023, foram analisadas 2.200.796 fichas de SRAG por COVID-19 no Brasil, sendo 1.198.507 em 2020, 4.294.934 em 2021, 1.568.476 em 2022 e 161.459 em 2023. Observou-se predominância de variáveis classificadas com completude muito ruim ao longo de todo o período analisado, embora com redução progressiva de 38,1% em 2020 para 30,3% em 2023. Em contrapartida, as variáveis classificadas com grau de completude bom aumentaram de 7,9 para 12,1% no mesmo período (Tabela 1). O maior volume de notificações ocorreu em 2021, seguido de redução acentuada nos anos subsequentes; contudo, persistiram elevadas proporções de incompletude nos registros.

**Tabela 1.** Percentual de variáveis dos casos de Síndrome Respiratória Aguda Grave por COVID-19 segundo grau de completude por ano de notificação no Brasil, 2020-2023.

Grau de completude	2020		2021		2022		2023	
	%	n (63)	%	n (66)	%	n (66)	%	n (66)
Excelente	17,5	11	18,2	12	16,7	11	15,2	10
Bom	7,9	5	3,0	2	9,1	6	12,1	8
Regular	25,4	16	19,7	13	19,7	13	18,2	12
Ruim	11,1	7	22,7	15	22,7	15	24,2	16
Muito ruim	38,1	24	36,4	24	31,8	21	30,3	20

Fonte: SIVEP-Gripe, 29/01/2024.

Os estados do Amapá, Mato Grosso do Sul, Santa Catarina e Paraná apresentaram as maiores proporções de variáveis classificadas como bom e excelente, alcançando em conjunto aproximadamente 40 a 45% de preenchimento satisfatório. Em contrapartida, Tocantins, Pernambuco, Mato Grosso, Rondônia e Alagoas exibiram os piores desempenhos, com predomínio das categorias ruim e muito ruim, superando 50%. No panorama nacional, observou-se que cerca de 30% das variáveis permaneceram em níveis insatisfatórios (ruim e muito ruim) (Figura 1).

**Figura 1.** Percentual de variáveis dos casos de Síndrome Respiratória Aguda Grave por COVID-19 segundo grau de completude e Unidade Federativa de residência, Brasil, 2020-2023.

Fonte: SIVEP-Gripe, 29/01/2024.

Na análise da oportunidade (Figura 2), verificou-se redução significativa nos intervalos médios de processamento dos registros. O tempo entre o início dos sintomas e a notificação diminuiu de 13 para 7 dias. De forma semelhante, os intervalos médios para digitação e encerramento reduziram-se em mais de 50%, evidenciando maior agilidade no fluxo do registro e na finalização das fichas.

**Figura 2.** Média do intervalo de tempo (em dias) das notificações, digitações e do encerramento da Síndrome Respiratória Aguda Grave por COVID-19 no Brasil, 2020-2023.

Fonte: SIVEP-Gripe, 29/01/2024.

A Figura 3 apresenta o percentual de registros notificados, digitados e encerrados oportunamente segundo as UF do Brasil. Em 2020, apenas 50% dos registros de SRAG por COVID-19 foram notificados no país no prazo de sete dias a partir da data do início dos sintomas. Em 2023, esse percentual aumentou para 76,9%. De forma semelhante ao padrão nacional, todos os estados apresentaram melhora na notificação oportuna. Apesar dos avanços, nenhuma UF atingiu a meta de 90% dos casos notificados no prazo recomendado.

**Figura 3.** Percentual de notificação, digitação e encerramento oportunos dos casos de Síndrome Respiratória Aguda Grave por COVID-19 segundo Unidade Federativa do Brasil, 2020-2023.

Fonte: SIVEP-Gripe, 29/01/2024.

A avaliação da oportunidade de digitação no Brasil revelou que, em 2020, mais de 87% das fichas foram digitadas em até 30 dias após a data de notificação, passando para 93% em 2023. A maioria dos estados aumentou a proporção de digitação oportuna ao longo dos anos, alcançando a meta de 90% estabelecida pelo Ministério da Saúde. No entanto, Espírito Santo, Mato Grosso, Pará, Pernambuco e Rio de Janeiro foram os estados que não atingiram esse parâmetro (Figura 3).

Quanto ao encerramento oportuno em até 60 dias da data da notificação, observou-se, durante o período analisado, que cerca de 86% dos casos foram encerrados oportunamente no Brasil, com pouca alteração entre os anos. Os estados que encerraram mais de 90% dos casos oportunamente foram Acre, Amapá, Mato Grosso do Sul, Paraíba, Piauí, Roraima, Santa Catarina e Sergipe. Em relação ao tempo de oportunidade, somente a meta de digitação oportuna foi alcançada nos anos de 2022 e 2023, enquanto as demais permaneceram abaixo dos parâmetros estabelecidos (Figura 3).

## DISCUSSÃO

O presente estudo analisou a completude e o tempo de oportunidade dos registros de SRAG por COVID-19 do SIVEP-Gripe. Constatou-se que a completude foi avaliada como insatisfatória, com 59% das variáveis classificadas como ruim e muito ruim. Em contrapartida, ocorreu uma significativa redução no tempo de notificação, digitação e encerramento das fichas, indicando uma maior agilidade no processo de alimentação do sistema. Esses achados corroboram estudos anteriores^3^ ^,^ ^12^, que também identificaram baixa completude nos registros de SRAG. Embora a redução no número de registros em 2022 e 2023 tenha acelerado os processos de notificação e encerramento, não houve avanço proporcional na qualidade do preenchimento, sugerindo que a agilidade operacional não foi acompanhada por melhorias na consistência dos dados.

A heterogeneidade observada entre as unidades federativas, com estados como Amapá, Mato Grosso do Sul, Santa Catarina e Paraná apresentando melhores níveis de completude, enquanto outros como Tocantins, Pernambuco e Rondônia apresentaram desempenho insatisfatório, sugere desigualdades estruturais na organização da vigilância epidemiológica. Essas diferenças podem refletir variações na infraestrutura tecnológica, na capacitação das equipes e nos fluxos operacionais de registro, incluindo o uso de fichas em papel com posterior digitação. Estudos prévios também apontam que desigualdades regionais impactam diretamente a qualidade dos sistemas de informação em saúde^7^. Cabe destacar que o SIVEP-Gripe é um sistema complexo, com múltiplas variáveis e instabilidades operacionais, que passou por sucessivas atualizações durante a pandemia, o que pode ter impactado a qualidade dos registros. Além disso, o acesso ao sistema não é universal entre os serviços de saúde, podendo influenciar a oportunidade das notificações.

A baixa completude pode estar associada a fatores operacionais e organizacionais, como a sobrecarga de trabalho durante os períodos críticos da pandemia, a priorização de atividades assistenciais em detrimento do registro detalhado das informações e a percepção da notificação como uma tarefa burocrática. Além disso, a falta de compreensão sobre a importância epidemiológica de determinadas variáveis pode contribuir para o preenchimento incompleto das fichas^15^. Esses fatores tornam-se ainda mais relevantes ao considerar o aumento expressivo das notificações entre 2020 e 2021, período em que o sistema foi fortemente demandado.

Em relação à oportunidade, observou-se redução significativa no tempo médio entre o início dos sintomas e a notificação, passando de 13 para 7 dias entre 2020 e 2023, além da melhoria nos intervalos de digitação e encerramento. Esses achados indicam adaptação progressiva dos serviços de vigilância epidemiológica ao longo da pandemia, com maior agilidade no processamento das informações. No entanto, apesar dessa evolução, a meta de 90% de notificações oportunas não foi atingida em nenhuma unidade federativa, evidenciando limitações persistentes na capacidade de resposta oportuna do sistema.

De forma semelhante, embora a digitação tenha alcançado percentuais superiores a 90% em grande parte dos estados nos anos mais recentes, o encerramento dos casos permaneceu em torno de 86%, sem atingir o parâmetro recomendado. Esse resultado sugere que etapas mais complexas do processo, como a investigação e a finalização dos casos, continuam sendo um desafio para os serviços de vigilância, mesmo após a consolidação do sistema ao longo da pandemia.

No início da crise sanitária, a gestão da pandemia exigia respostas rápidas e a adaptação dos SIS para coleta, análise e disseminação de dados em tempo real, essenciais à compreensão do cenário epidemiológico e à tomada de decisão. Para isso, os dados deveriam ser completos, confiáveis e oportunos^18^ ^,^ ^19^.

Durante os primeiros anos da pandemia, o atraso nos registros pode ter sido agravado por limitações do SIVEP-Gripe, que inicialmente não contemplava campos específicos para a COVID-19, além de instabilidades frequentes e ausência de interoperabilidade com outros sistemas, como o Sistema Gerenciador de Ambiente Laboratorial. Soma-se a isso a carência de capacitação sistemática dos profissionais responsáveis pelo preenchimento das fichas^7^.

A baixa qualidade de dados evidenciada pode estar relacionada a episódios de indisponibilidade ocorridos no acesso aos dados do SIVEP-Gripe, conhecidos como “apagões de dados”. Destaca-se a interrupção do painel Coronavírus em junho de 2020, por motivos políticos, bem como nova indisponibilidade em dezembro de 2021, que perdurou por ao menos 30 dias. Nesse contexto, o governo federal propôs alterações arbitrárias nas medições de variáveis e padrões de relatórios, em desacordo com as diretrizes epidemiológicas internacionais^20^. Esses eventos podem comprometer a produção de indicadores confiáveis, a capacidade de resposta rápida, além de dificultar a identificação de eventos relevantes e prejudicar a formulação de recomendações adequadas^21^.

Este estudo permitiu uma análise abrangente da qualidade de dados de COVID-19 no Brasil ao longo de quatro anos de pandemia, proporcionando subsídios para o aprimoramento dos registros no SIVEP-Gripe. Embora este estudo revele achados importantes para a vigilância e avaliação da qualidade da informação em saúde, ele apresenta limitações, como a análise restrita aos casos confirmados, em detrimento do conjunto total de casos registrados no sistema; além da ausência de limpeza do banco para eliminação de duplicidades e inconsistências, devido à falta de acesso aos dados individuais.

Como ponto forte, destaca-se a originalidade da análise conjunta da completude e da oportunidade dos registros, fornecendo evidências que podem subsidiar propostas de melhoria no preenchimento das fichas e no funcionamento do sistema. Um SIVEP-Gripe mais qualificado e oportuno contribui para o monitoramento eficaz do território e para decisões mais assertivas em saúde pública.

No âmbito dos SIS, registros, processamento e análise de dados ainda enfrentam desafios. Segundo Melo e Soares^6^, existem dificuldades relacionadas aos SIS, como a ausência de uma política de incentivo ao registro correto dos dados, dificultando a geração de informações corretas e, consequentemente, a realização de ações eficazes^22^.

Os resultados indicam que, embora tenham ocorrido avanços na agilidade dos processos, estes não foram acompanhados por melhorias consistentes na qualidade do preenchimento. Isso reforça a necessidade de implementação de rotinas periódicas de avaliação dos registros, bem como de estratégias de qualificação da informação nos serviços de vigilância epidemiológica.

Apesar das limitações observadas, o SIVEP-Gripe configura-se como um sistema fundamental para a vigilância epidemiológica, especialmente em situações de emergência. Por ser um dos poucos sistemas online com acesso rápido aos dados, permite análises oportunas e subsidia a tomada de decisão. Essa característica o diferencia de outros sistemas, como o Sinan Net, que, embora igualmente relevante, apresenta fluxos menos ágeis de disponibilização das informações.

Além disso, é fundamental instituir processos contínuos de capacitação dos profissionais envolvidos na notificação, visando garantir a padronização e a confiabilidade das informações. Políticas de capacitação contínua, avaliações periódicas, supervisão ativa e monitoramento sistemático da qualidade da informação são fundamentais para reduzir as disparidades regionais e assegurar dados confiáveis, indispensáveis ao planejamento de ações em saúde coletiva.
